# TRANSNAP: a web database providing comprehensive information on Japanese pear transcriptome

**DOI:** 10.1038/s41598-019-55287-4

**Published:** 2019-12-12

**Authors:** Shizuka Koshimizu, Yukino Nakamura, Chikako Nishitani, Masaaki Kobayashi, Hajime Ohyanagi, Toshiya Yamamoto, Kentaro Yano

**Affiliations:** 1 School of Agriculture, Meiji University, Kawasaki, 214-8571 Japan; 2 National Agriculture and Food Research Organization (NARO), Tsukuba, 305-8517 Japan; 3 King Abdullah University of Science and Technology (KAUST), Computational Bioscience Research Center (CBRC), Thuwal, 23955-6900 Saudi Arabia

**Keywords:** Plant molecular biology, Transcriptomics

## Abstract

Japanese pear (*Pyrus pyrifolia*) is a major fruit tree in the family Rosaceae and is bred for fruit production. To promote the development of breeding strategies and molecular research for Japanese pear, we sequenced the transcripts of Japanese pear variety ‘Hosui’. To exhaustively collect information of total gene expression, RNA samples from various organs and stages of Japanese pear were sequenced by three technologies, single-molecule real-time (SMRT) sequencing, 454 pyrosequencing, and Sanger sequencing. Using all those reads, we determined comprehensive reference sequences of Japanese pear. Then, their protein sequences were predicted, and biological functional annotations were assigned. Finally, we developed a web database, TRANSNAP (http://plantomics.mind.meiji.ac.jp/nashi), which is the first web resource of Japanese pear omics information. This database provides highly reliable information via a user-friendly web interface: the reference sequences, gene functional annotations, and gene expression profiles from microarray experiments. In addition, based on sequence comparisons among Japanese, Chinese and European pears, similar protein sequences among the pears and species-specific proteins in Japanese pear can be quickly and efficiently identified. TRANSNAP will aid molecular research and breeding in Japanese pear, and its information is available for comparative analysis among other pear species and families.

## Introduction

Pears (*Pyrus* spp.) are deciduous trees of the genus *Pyrus* in the family Rosaceae. Pears are economically important fruit trees, having the third largest production of fruits in the world. Among thousands of species in *Pyrus*, 22 primary species in the genus proposed by Bell *et al*.^1^ are well known. Furthermore, only a few of the 22 primary species, including Japanese pear (*P. pyrifolia*), Chinese pear (*P. bretschneideri*), Siberian pear (*P. ussuriensis*), and European pear (*P. communis*), have been grown for fruit production^2^.

Currently, significant amounts of genomic information on pears have been published: draft sequences (scaffolds) of the Chinese pear genome based on a combination of BAC-to-BAC and Illumina HiSeq strategy^3^, chromosomal-level sequences (pseudomolecules) of the Chinese pear genome^4^, and the genome sequence of European pear obtained by 454 pyrosequencing^5^. This information is available from the Pear Genome Project (http://peargenome.njau.edu.cn) and the Genome Database for Rosaceae^6^ (GDR; http://www.rosaceae.org/). The Pear Genome Project provides information on scaffold sequences of the Chinese pear assembly and structural annotations of gene and protein sequences. The GDR provides information on genome sequences, gene models, gene functional annotations with gene ontology (GO) terms^7,8^, DNA markers, genetic maps, and orthologs and syntenies in the Rosaceae, including Chinese and European pears. Besides sequence data in NCBI^9^, the GDR also provides portal pages to access its sequence data and sequence read archive (SRA). In addition, metabolome analyses were conducted in European pear and the metabolites related to fruit development were identified^10,11^. The information on Chinese and European pears is considerably useful for breeding and research on Japanese pear. However, Japanese pear has some distinctive features; for example, non-climacteric maturation^12^, round-shaped fruits, a water core, gibberellin promotion of fruit expansion^13^, and species-specific susceptibility to pests and pathogens^14^. Therefore, improved data on Japanese pear will lead to advances in molecular breeding and research. Here, we analyzed Japanese pear variety ‘Hosui’ (syn. ‘Housui’), which is one of the most widely cultivated varieties and is used for crossbreeding because of its excellent texture and taste^14^.

To obtain reliable omics information on Japanese pear, we conducted comprehensive sequencing and analysis. Pacific BioSciences provides more robust sequence data than second-generation sequencers by using longer sequencing techniques^15,16^, especially in highly heterogeneous species^17^, including pears. Therefore, we generated PacBio Iso-Seq data obtained from various organs and stages in leaves, flowers, and fruits, and constructed high quality (HQ) full-length cDNAs. In addition, we constructed transcriptome contigs by hybrid assembly of 454 and Sanger sequencing data. Then, the HQ full-length cDNAs and transcriptome contigs were integrated into a comprehensive catalog of transcripts, which we call reference sequences here, for Japanese pear variety ‘Hosui’.

Finally, we developed a public web resource, the Japanese Pear Transcriptome Database (TRANSNAP; http://plantomics.mind.meiji.ac.jp/nashi). In this database, users can freely access the reference sequences of the transcriptome and their functional annotations with a user-friendly graphical user interface. Moreover, gene expression profiles from microarray experiments are easily searchable and browsable in TRANSNAP. This will facilitate molecular research and breeding in pears.

## Results

### Reference sequences of transcripts in Japanese pear

RNA samples for sequencing were prepared from each organ of leaves, flowers, and fruits of Japanese pear variety ‘Hosui’ (Supplementary Table 1). We performed single-molecule real-time (SMRT) sequencing (Pacific BioSciences), Pyrosequencing (Roche 454), and Sanger sequencing (Applied Biosystems) (Supplementary Table 2). For the sequencing, a total of 9 Gbp of PacBio reads, 612 Mbp of 454 reads, and 24 Mbp of Sanger reads were obtained from the libraries. For PacBio reads, a total of 56,331 HQ full-length cDNAs were generated on the pipeline of Iso-Seq. The 454 and Sanger reads were pre-processed, and the remaining 395 Mbp reads were used for assembly. As a result, a total of 43,963 contigs were generated by Newbler (Roche Diagnostics Corporation), and redundant contigs with the PacBio HQ full-length cDNA were removed. Then, 49,866 non-redundant transcript sequences were obtained. Finally, short sequences (< 200 bp) were eliminated, and the remaining 47,202 sequences were defined as the reference sequences of the Japanese pear transcriptome (Table 1).

**Table 1:** A summary of reference sequences.

Number of reference sequences	47,202
Total length (bp)	57,512,278
N50 (bp)	1,763
Average length (bp)	1,218
Number of genes (loci)	41,221
Number of predicted proteins	44,098
Number of protein-coding sequences from a start codon to a stop codon	23,239

### Prediction of protein sequences from reference sequences

From the 47,202 transcripts (reference sequences), a total of 44,098 (93%) protein sequences were predicted in 38,687 loci using TransDecoder^18^ (Table 1). Respectively, 22,494 and 21,604 of the 44,098 predicted protein sequences were obtained from the HQ full-length cDNAs and contigs. Among them, protein sequences completely predicted from a start codon to a stop codon were 15,977 out of 22,494 (71%) and 7,262 out of 21,604 (33.6%) in the HQ full-length cDNAs and contigs, respectively (Table 1). The higher completion rate in the HQ full-length cDNAs indicates the advantage of SMRT sequencing for the prediction of full-length cDNAs. Out of the 47,202 transcripts, the 3,104 transcripts were not predicted proteins due to the short lengths of contigs (average length, 293.1 bp).

### Functional annotations

Based on a BLASTP similarity searches (e-value < 1e-5)^19,20^ against two major protein databases, the NCBI non-redundant protein database (nr) and Swiss-Prot in UniProt^21^, we assigned functional annotations to 37,400 (84.8%) and 29,730 (67.4%) predicted protein sequences out of 44,098, respectively. In addition, 36,036 (81.7%) protein sequences were assigned with functional domains and GO terms by InterProScan^22^, and 10,871 (24.7%) protein sequences were annotated with KEGG^23^ Orthology by KAAS^24^.

### Identification of species-specific proteins in Japanese pear

Based on BLASTP searches (e-value < 1e-3), we selected Japanese pear protein sequences having no similar sequence in sequence databases of Chinese and European pears. As a result, 5,850 protein sequences were defined as species-specific proteins in Japanese pear (Supplementary Table 3). Out of the 5,850 protein sequences, 349 were assigned with GO terms. The top three of GO terms (biological process) from the GO enrichment analysis were ‘transport’ (GO:0006810, 14 proteins), ‘oxidation-reduction process’ (GO:0055114, 10 proteins), and ‘regulation of transcription, DNA-templated’ (GO:0006355, 9 proteins). By using the same method, we defined species-specific proteins in Chinese (270 proteins) and European pears (3,264 proteins). The top three of GO terms (biological process) from the GO enrichment analysis were ‘N-acetylglucosamine metabolic process’ (GO:0006044, 16 proteins), ‘carbohydrate metabolic process’ (GO:0005975, 11 proteins), and ‘translation’ (GO:0006412, 8 proteins) in Chinese pear, and ‘proteolysis’ (GO:0006508, 10 proteins), ‘DNA recombination’ (GO:0006310, 8 proteins), and ‘double-strand break repair’ (GO:0006302, 5 proteins) in European pear.

### Database contents and functions

**Figure 1:**
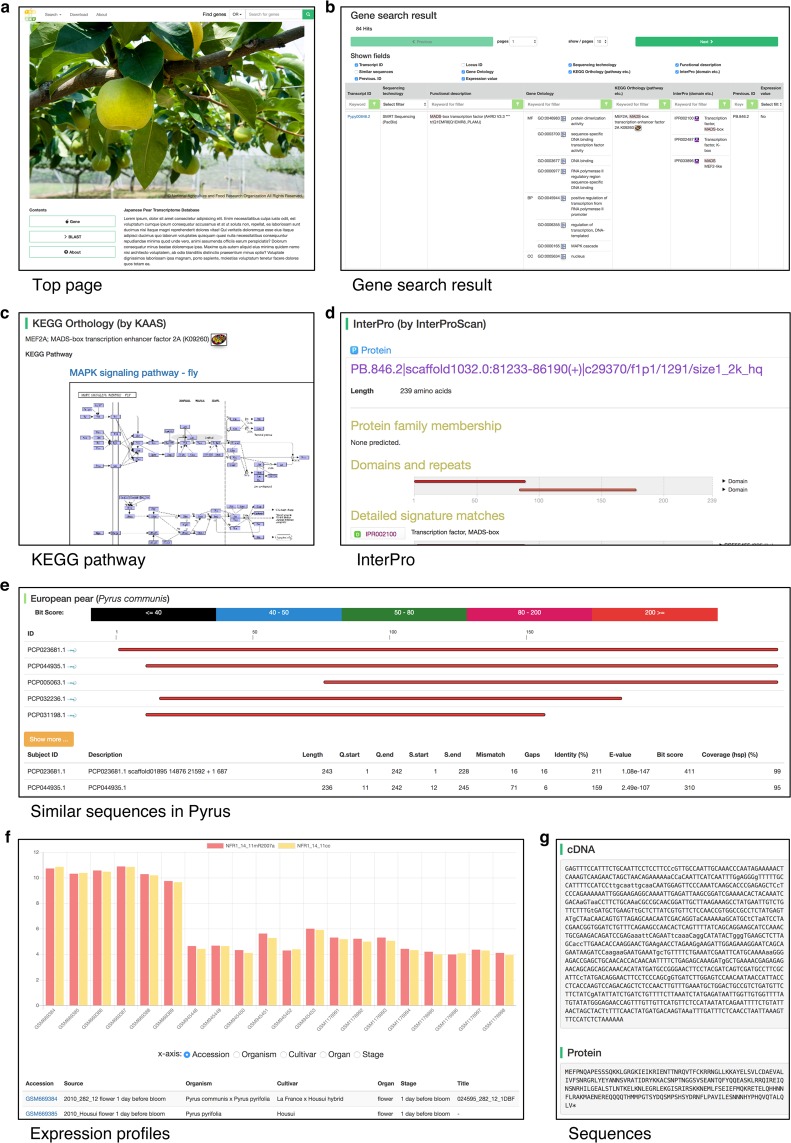
Schematic representation and screen shots of the Japanese pear transcriptome database TRANSNAP. (**a**) Screen shots of the top page. (**b**) Gene search result page. The information of each transcript is shown in a web page. It contains (**c**) example data of the KEGG^23^ pathway obtained by the KEGG API (please see Materials and Methods), (**d**) InterPro, (**e**) similar sequences in *Pyrus*, (**f**) expression profiles, and (**g**) cDNA and protein sequences.

We developed the Japanese pear transcriptome database TRANSNAP and stored analyzed data of transcripts, protein sequences, and their annotations. Users can search genes in TRANSNAP in two ways, a keyword search and a BLAST search from the top page (Fig. 1a). In the keyword search, any of the transcript IDs, functional descriptions, GO terms, domains, and metabolic pathways are searchable. In the BLAST search, users can search similar transcripts or protein sequences using nucleotide or protein query sequences. A result page with these search functions shows the retrieved records in a table format (Fig. 1b). In this table, records of interest can be extracted by the on-the-fly filtering function in each field (column). The details page for each gene is comprised of several sections: Summary, Functional annotations, Similar sequences in *Pyrus*, Expression profiles, and Sequences (Fig. S1).

In the details page, the ‘Summary’ section provides information on the locus ID, gene description, sequencing method, and summary of computational annotations by GO, KEGG, InterPro, and BLAST. In the ‘Functional annotation’ section, metabolic pathways by KEGG (Fig. 1c), protein families and functional domains by InterPro (Fig. 1d), and similar sequences obtained by BLASTP against the NCBI nr and Swiss-Prot in UniProt databases are shown. The ‘Similar sequences in *Pyrus*’ section shows similar protein sequences in Chinese and European pears to the Japanese pear protein sequences obtained by BLASTP (Fig. 1e). This information allows us to identify species-specific proteins in Japanese pear. In the ‘Expression profiles’ section, gene expression data from microarray experiments is explored in graphs and tables (Fig. 1f). The expression pattern is shown as bar graphs. The sample IDs (GSM IDs) in the table have hyperlinks to jump to the original page in the NCBI GEO^25^ website. The genome browser JBrowse^26^ provides information on the positions of microarray probes in the reference sequences of Japanese pears in the subsection ‘JBrowse for microarray probes’. In the ‘Sequences’ section, cDNAs and their protein sequences are obtained (Fig. 1g).

### An example of a gene search in TRANSNAP

Flowering is one of the key physiological mechanisms for fruit production. By using TRANSNAP, here we can explore genes involved in flowering. The keyword ‘flowering’ is entered in the search box of the ‘Gene Search’ page. By clicking the ‘Search’ button, a search result with 642 transcripts is retrieved within several seconds. To examine gene expression profiles, genes having expression information are selected by using the pull-down menu in the column ‘expression value’ in the table. For the selection, ‘Yes’ in the pull-down menu is selected, then 129 transcripts remain. As an example of a search result, the information for transcript ‘Pypy01331.1’ is introduced here. By clicking the hyperlink of the transcript ID, a new window is shown that provides detailed information (Fig. S1). In the details page, annotations in ‘Description’, ‘InterPro’, ‘Gene Ontology’, and ‘KEGG Orthology’ sections strongly suggest that this transcript plays a role as a MADS-box transcription factor. From the BLASTP annotation with the NCBI nr database, the similar sequence of the MADS-box protein in Japanese pear (Acc. AJW29041) is found. BLASTP annotations with the Swiss-Prot in UniProt database provide similar proteins of MADS-box transcription factors in *Arabidopsis thaliana*, *Petunia hybrida*, and *Solanum lycopersicum*. A similar MADS-box transcription factor protein in Chinese pear (rna10647) is identified with a BLASTP search. Although a similar sequence in European pear (PCP023681.1) is identified, annotation is not assigned. Both the proteins of Chinese and European pears may be counterparts of the transcript in Japanese pear (Pypy01331.1). According to gene expression data, this transcript is highly expressed in flowers compared to other organs. In addition, sequence data of the cDNA and protein in TRANSNAP, the coding sequence from the start codon to the stop codon and the complete protein sequence (Met to *, the symbol * means a stop codon) are browsable.

## Discussion

‘TRANSNAP’ is the first database that provides annotation information on transcriptome data in Japanese pear. We aimed to exhaustively collect information of expressed genes, thereby obtaining high quality cDNA sequences. For a comprehensive analysis, we obtained reference sequences of the Japanese pear transcriptome by integration of reads from various organs and stages using three types of sequencing technology (Supplementary Table 1). Given the combined sequencing approaches and origins of RNA samples, the reference sequences are considered to cover nearly all expressed gene sequences. Out of the 44,098 protein-coding sequences from 38,687 loci, 23,239 protein-coding sequences that begin with start codons and end at stop codons were identified in 20,060 loci (Table 1). With the 23,239 protein-coding sequences, cDNAs including transcript variants and protein sequences were predicted. The representative protein sequences in each locus were compared with *A. thaliana* protein sequences by BLASTP (≤ 1e-10 e-value and ≥ 80% alignment coverage of the Japanese pear protein sequences) to validate the accuracy of the predicted protein sequences. As a result, the 14,748 protein sequences in Japanese pear cover the complete *A. thaliana* protein sequences. The average identities of the aligned regions between the 14,748 Japanese pear protein sequences and *A. thaliana* protein sequences was 67.0%. A comparison of the average identities between two pear species (Chinese and European pears) and *A. thaliana* protein sequences showed average identities of 60.9% and 59.5%, respectively. Therefore, the 14,748 Japanese pear protein sequences had the highest average identity (67.0%). Among 14,748 protein sequences in Japanese pear, 12,664 protein sequences (85.9%) showed equal or greater than 50% identity (Fig. S1). Surprisingly, identities higher than 70% were found for alignments of the 6,810 Japanese pear protein sequences (46.2%). Thus, the fact that the 23,239 complete protein sequences of Japanese pear detected in this study contain 14,748 highly conserved proteins with *A. thaliana* in spite of being genetically divergent taxa (i.e. orders Rosales and Brassicales) demonstrates the benefits of our approach and the reliability of the results as provided from the TRANSNAP database.

We provide information on highly reliable reference sequences for Japanese pear in the TRANSNAP database. This database also contains information on gene functional annotations, gene expression data from microarray experiments, and similar protein sequences in other pear species. With the search function in TRANSNAP, users can easily access information on genes related to physiological mechanisms such as fertilization, fruit maturation, and other specific phenomena in Japanese pear. We integrated omics information from Japanese pear and provide it via a user-friendly web interface.

Comparative analyses among plant species facilitate breeding strategies for the improvement of various key agronomical traits such as plant growth, photosynthesis, flowering and fertilization, yield, quality, fruit nutrient content, and defense against disease and pests. However, Comparative analyses among species of the *Pyrus* genus or Rosaceae are not available in the current version of TRANSNAP. Therefore, we are currently planning the interoperability of TRANSNAP with the ‘Plant Omics Data Center’ (PODC, http://plantomics.mind.meiji.ac.jp/podc/) database^27,28^, which provides knowledge-based annotations of gene functions, cis-elements, transcription factors, gene expression networks, and orthologs among model plant species and crops. By using both the TRANSNAP and PODC databases together, information on specific genes in Japanese pear and their orthologs in other plant species and molecular functions, including regulation of gene expression, will be easily accessible. Along with additional comparative omics information between Japanese pear and other pear species, we will update TRANSNAP in conjunction with PODC in the future.

We have constructed and are maintaining the first omics database, TRANSNAP, for Japanese pear. The highly reliable information of the reference sequences, gene expression, and comprehensive annotations provided in TRANSNAP will be a key online resource for *Pyrus*. Furthermore, the comparative omics information can be applied to design breeding approaches among *Pyrus* or Rosaceae, as well as to much more distantly-related species.

## Materials and Methods

### Plant materials

Trees of Japanese pear (*Pyrus pyrifolia* Nakai) ‘Housui’ (syn. ‘Hosui’) were managed according to a standard orchard system used at the Institute of Fruit Tree and Tea Science, NARO. RNA samples were separately collected (Supplementary Table 1), frozen in liquid nitrogen, and stored at −80 °C. Total RNAs were extracted as previously described^29^. RNA concentration and integrity were evaluated with an ND-1000 spectrophotometer (LMS) and an Agilent 2100 Bioanalyzer (Agilent Technologies).

### Construction of sequencing libraries and sequencing methods

For SMRT sequencing, cDNA libraries for full-length isoform sequencing were constructed under size fractions (3-6 kb, 2-3 kb, 1-2 kb) using the standard SMRT method (Pacific BioSciences). Sequencing was performed on the Pacific Biosciences RS II sequencer. For 454 pyrosequencing, a normalized full-length-enriched cDNA library was constructed by DNAFORM Inc. using the cap-trapper technique^30^. Shotgun sequencing, 5′-end sequencing, and 3′-end sequencing libraries were constructed using a GS FLX Titanium Rapid Library Preparation Kit (Roche Diagnostics Corporation). Molecular Identifier (MID) tags, RL1 (ACACGACGACT), RL2 (ACACGTAGTAT), and RL3 (ACACTACTCGT), were added at the 5′ ends of the inserts in each shotgun sequencing, 5′-end sequencing, and 3′-end sequencing libraries, respectively. Sequencing was performed by 454 GS-FLX Titanium technology (Roche Diagnostics Corporation) for the libraries. Sanger sequencing and the cDNA library construction were performed as reported by Nishitani *et al*. (2010) (accession number: DB999954-DB999984)^31^.

### Construction of high quality (HQ) full-length cDNAs

**Figure 2:**
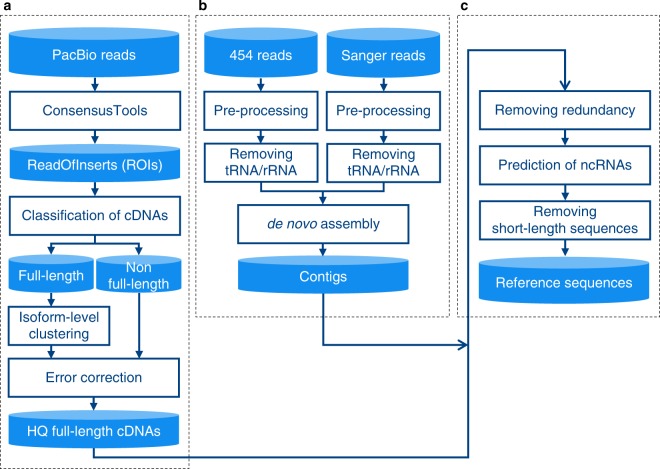
Workflow for the construction of reference sequences of the *Pyrus pyrifolia* transcriptome. (**a**) PacBio reads were analyzed using the pipeline Iso-Seq. (**b**) Pre-processed Sanger and 454 reads were hybrid-assembled with Newbler. (**c**) HQ full-length cDNAs and contigs were integrated. Redundant sequences and short sequences (< 200 bp) except for ncRNAs were filtered, and reference sequences were generated.

HQ full-length cDNAs were generated following the RS_IsoSeq protocol (SMRT Analysis 2.3). By using ConsensusTools in SMRT Analysis with parameters ‘-minFullPasses 0’ and ‘-minPredictedAccuracy 75’, ReadsOfInserts (ROIs) were obtained from PacBio reads. Then, the ROIs were classified as either full-length cDNAs or partial cDNAs by ‘pbtranscript.py classify’ in SMRT Analysis. Isoform-level clustering was performed to cluster the full-length cDNAs and remove sequence redundancy by each size (3-6 kb, 2-3 kb, 1-2 kb) using ‘pbtranscript.py cluster’ in SMRT Analysis. At the same time, sequencing errors in the full-length and partial cDNAs were corrected using the option ‘quiver’, then HQ full-length cDNAs were generated (Fig. 2a).

### Pre-processing of 454 pyrosequencing reads

The 454 reads were pre-processed by removing duplicate reads using CLC Genomics Workbench (version 7.0; Qiagen) and trimming adapters and poly-A sequences using Cutadapt^32^ (version 1.9.1). Low quality sequences were removed using an in-house Perl script. The pre-processing reads were searched with BLASTN (e-value < 1e-5) against rRNA and tRNA databases, which were obtained from TAIR10^33^ and RAP-DB^34^. Reads having similar sequences with rRNA and tRNA were removed (Fig. 2b). The remaining reads were used for constructing contigs.

### Pre-processing of Sanger sequencing reads

Sanger reads were analyzed by Phred^35^ (version: 0.071220.b), and vector and low quality regions in sequences were removed using Cross_match^36^ (version 1.08721) and an in-house Perl script, respectively. The reads derived from rRNA and tRNA were removed by the same method as for the procedure for the 454 reads (Fig. 2b). The remaining reads were used for construct contigs.

### Construction of contigs

The pre-processed 454 and Sanger reads were assembled into contigs using Newbler (Roche Diagnostics Corporation) with the ‘urt’ option (Fig. 2b).

### Construction of reference sequences

We integrated and removed redundant sequences using in-house scripts and CD-HIT-EST^37,38^ (version 4.6) with the parameter ‘-c 0.99’. We removed short-length sequences (< 200 bp) except for sequences assigned an annotation of non-cording RNA (ncRNA) (see ‘Functional annotations’ section). The threshold length for removing sequences (< 200 bp) was empirically determined by referring to the distributions of sequence lengths of coding sequences in *Arabidopsis*, rice, Chinese pear, and European pear. The remaining sequences were used as reference sequences of Japanese pear for further analysis (Fig. 2c).

### Prediction of protein sequences

Open reading frames (ORFs) were predicted using TransDecoder (version 3.0.0) with ‘-m 30 -S’ and ‘-m 30’ parameters for the HQ full-length cDNAs and contigs, respectively. The similarity of these ORFs to known protein sequences was examined by executing BLASTP and HMMER^39^ (version 3.1b1) with the TAIR10 and Pfam databases^40^, respectively. Using the results of the similarity searches, coding regions and protein sequences were predicted by TransDecoder with the following parameters:-single_best_orf, -retain_blastp_hits, and -retain_pfam_hits. When protein sequences were not predicted, the coding regions and protein sequences were predicted by TransDecoder without the result of the similarity searches.

### Functional annotations

Sequence similarity searches of predicted protein sequences were performed by BLASTP searches (e-value < 1e-5) against the NCBI nr database and Swiss-Prot. In addition, KEGG^23^ Orthology were assigned to each transcript by the web tool KAAS^24^ with eudicot and monocot datasets. In our database TRANSNAP, a web page for the information of each transcript contains functional descriptions including the KEGG Orthology and KEGG pathway maps. In displaying the KEGG pathway maps in the web page, the KEGG API available from the KEGG web site is executed. The KEGG API allows us to easily and quickly obtain the latest information of the KEGG pathway maps for each KEGG Orthology. Functional analysis of the protein sequences was performed using InterProScan. Prediction of ncRNA was performed using Infernal^41^ (version 1.1.2) with the Rfam database^42,43^.

### Gene expression profiles

Microarray experimental data for Japanese pear were downloaded from the GEO repository database. For the microarray platform GPL13124, expression data (GSE27090, GSE38550, and GSE48393) were obtained. Expression data (GSE18682, GSE27090, and GSE34845) with the platform GPL9476 were also obtained. The microarray data were normalized by the quantile method using the library limma^44^ of R software (https://www.r-project.org/). Each probe sequence was aligned to the reference sequences using BLASTN (e-value < 1e-10).

### Similar protein sequences in *Pyrus*

We executed BLASTP searches (e-value < 1e-3) for predicted protein sequences of Japanese pear against Chinese and European pear protein sequences^4,5^. Similar protein sequences against each pear were obtained.

### Database construction

The TRANSNAP database was implemented on a Linux CentOS (version 6.8) with an Apache web server (version 2.2.15) and MySQL database server (version 5.6). PHP (version 5.6) and JavaScript were used for the server-side processing and the client-side processing, respectively. For rich user interface applications, JavaScript libraries (Vue [https://jp.vuejs.org/], Bootstrap [http://getbootstrap.com/], and Chart.js [http://www.chartjs.org/]) were employed.

## Supplementary information

Supplementary Information

Supplementary Table 1

Supplementary Table 2

Supplementary Table 3

## Data Availability

Both of the RNA-seq data using PacBio and Roche 454 have been deposited in the DDBJ Sequence Read Archive (DRA) (accession number DRA008208). The database TRANSNAP (http://plantomics.mind.meiji.ac.jp/nashi) developed in this study is freely available.
