# Supplementary material for: TRANSNAP: a web database providing comprehensive information on Japanese pear transcriptome
Source: Sci Rep. 2019 Dec 12;9:18922. doi: 10.1038/s41598-019-55287-4 (PMC6908688; doi:10.1038/s41598-019-55287-4)
Supplement: Supplementary file 1 — Supplementary Information [file 41598_2019_55287_MOESM1_ESM.pdf]

TRANSNAP: a web database providing comprehensive information on Japanese pear transcriptome

Shizuka Koshimizu[1,†], Yukino Nakamura[1,†], Chikako Nishitani[2], Masaaki Kobayashi[1], Hajime Ohyanagi[1,3], Toshiya Yamamoto[2,*], Kentaro Yano[1,*]

[1] School of Agriculture, Meiji University, Kawasaki 214-8571, Japan

[2] NARO Institute of Fruit Tree Science, Tsukuba 305-8605, Japan

[3] KAUST (King Abdullah University of Science and Technology), CBRC (Computational Bioscience Research Center), Thuwal, 23955-6900, Saudi Arabia

* Corresponding Author

† These authors contributed equally to this work.

Transcript ID: Pypy01331.1

Summary

Entry summary

**Locus ID**	Pypy01331
**Transcript ID**	Pypy01331.1
**Description**	Agamous MADS-box transcription factor (AHRD V3.3 *** tr|B2CDE2|B2CDE2_9ASPA)
**Sequence technology**	Sanger+Pyrosequencing
**cDNA length**	1096 bp

Annotation summary

**InterPro (domain etc.)**	IPR002100: Transcription factor, MADS-box (http://www.ebi.ac.uk/interpro/entry/IPR002100)<br>IPR002487: Transcription factor, K-box (http://www.ebi.ac.uk/interpro/entry/IPR002487)<br>IPR033896: MADS MEF2-like (http://www.ebi.ac.uk/interpro/entry/IPR033896)
**Gene Ontology**	GO:0000165: MAPK cascade (BP) (http://amigo.geneontology.org/amigo/term/MAPK cascade)<br>GO:0000977: RNA polymerase II regulatory region sequence-specific DNA binding (MF) (http://amigo.geneontology.org/amigo/term/RNA polymerase II regulatory region sequence-specific DNA binding)<br>GO:0003677: DNA binding (MF) (http://amigo.geneontology.org/amigo/term/DNA binding)<br>GO:0003700: sequence-specific DNA binding transcription factor activity (MF) (http://amigo.geneontology.org/amigo/term/sequence-specific DNA binding transcription factor activity)<br>GO:0005634: nucleus (CC) (http://amigo.geneontology.org/amigo/term/nucleus)<br>GO:0006355: regulation of transcription, DNA-templated (BP) (http://amigo.geneontology.org/amigo/term/regulation of transcription, DNA-templated)<br>GO:0045944: positive regulation of transcription from RNA polymerase II promoter (BP) (http://amigo.geneontology.org/amigo/term/positive regulation of transcription from RNA polymerase II promoter)<br>GO:0046983: protein dimerization activity (MF) (http://amigo.geneontology.org/amigo/term/protein dimerization activity)
**KEGG Orthology (pathway etc.)**	K09264: K09264; MADS-box transcription factor, plant (http://www.kegg.jp/dbget-bin/www_bget?ko+K09264; MADS-box transcription factor, plant)
**Most similar sequence in nr (BLAST tophit)**	transcription factor [Pyrus pyrifolia]
**Most similar sequence in Swiss-Prot (BLAST tophit)**	sp|P29381|AGL1_ARATH Agamous-like MADS-box protein AGL1 OS=Arabidopsis thaliana GN=AGL1 PE=1 SV=1

Splicing variants

Not predicted.

Functional Annotations

KEGG Orthology (by KAAS)

K09264; MADS-box transcription factor, plant (K09264) (http://www.kegg.jp/dbget-bin/www_bget?K09264)

InterPro (by InterProScan)

Protein

PB.1331.1|scaffold11.0:856029-864971(-)|isotig13304

Length 242 amino acids

Protein family membership

None predicted.

Domains and repeats

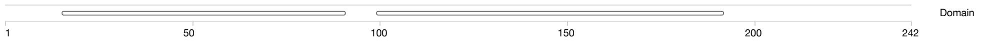

Detailed signature matches

IPR002100 Transcription factor, MADS-box (http://www.ebi.ac.uk/interpro/entry/IPR002100)

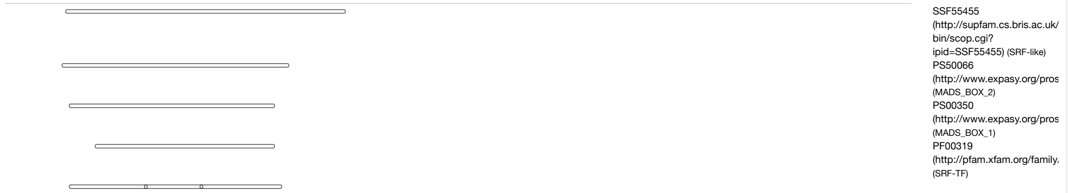

PR00404 (http://www.bioinf.manchest bin/dbbrowser/sprint/search prints_accn=PR00404&disp (MADSDOMAIN)
SM00432 (http://smart.embl-heidelberg.de/smart/do_ann ACC=SM00432&BLAST=DU (madsneu2)

D IPR033896	MADS MEF2-like (http://www.ebi.ac.uk/interpro/entry/IPR033896)	cd00265 (http://www.ncbi.nlm.nih.gov uid=cd00265) (MADS_MEF2_...)
D IPR002487	Transcription factor, K-box (http://www.ebi.ac.uk/interpro/entry/IPR002487)	PS51297 (http://www.expasy.org/pros (K_BOX) PF01486 (http://pfam.xfam.org/family, (K-box)
? no IPR	Unintegrated signatures	Coil (http://embnet.vital-it.ch/software/COILS_form.h PTHR11945 (http://www.pantherdb.org/p clsAccession=PTHR11945) (MADS BOX...) PTHR11945:SF150 (http://www.pantherdb.org/p clsAccession=PTHR11945:S (AG...) mobidb-lite (http://mobidb.bio.unipd.it/) (disord...)

Residue annotation

DNA binding site c...
dimerization inter...
putative phosphory...

GO term prediction

Biological Process

GO:0000165 (http://www.ebi.ac.uk/QuickGO/GTerm?id=GO:0000165) MAPK cascade
GO:0006355 (http://www.ebi.ac.uk/QuickGO/GTerm?id=GO:0006355) regulation of transcription, DNA-templated
GO:0045944 (http://www.ebi.ac.uk/QuickGO/GTerm?id=GO:0045944) positive regulation of transcription from RNA polymerase II promoter

Molecular Function

GO:0000977 (http://www.ebi.ac.uk/QuickGO/GTerm?id=GO:0000977) RNA polymerase II regulatory region sequence-specific DNA binding
GO:0003677 (http://www.ebi.ac.uk/QuickGO/GTerm?id=GO:0003677) DNA binding
GO:0003700 (http://www.ebi.ac.uk/QuickGO/GTerm?id=GO:0003700) transcription factor activity, sequence-specific DNA binding
GO:0046983 (http://www.ebi.ac.uk/QuickGO/GTerm?id=GO:0046983) protein dimerization activity

Cellular Component

GO:0005634 (http://www.ebi.ac.uk/QuickGO/GTerm?id=GO:0005634) nucleus

BLAST

Query:Protein

NCBI -nr-

Bit Score: <= 40 | 40 - 50 | 50 - 80 | 80 - 200 | 200 >=

ID 1 50 100 150

BAM71400 (https://www.ncbi.nlm.nih.gov/protein/BAM71400)

AJW29041 (https://www.ncbi.nlm.nih.gov/protein/AJW29041)

NP_001280918 (https://www.ncbi.nlm.nih.gov/protein/NP_001280918)

XP_008342376 (https://www.ncbi.nlm.nih.gov/protein/XP_008342376)

XP_017179299 (https://www.ncbi.nlm.nih.gov/protein/XP_017179299)

Subject ID	Description	Length	Q.start	Q.end	S.start	S.end	Mismatch	Gaps	Identity (%)	E-value	Bit score	Coverage (hsp) (%)
BAM71400	transcription factor [Pyrus pyrifolia]	242	1	242	1	242	1	0	241	3.14e-179	500	99
AJW29041	AG-like MADS-box protein [Pyrus pyrifolia]	243	1	242	1	243	4	1	238	2.36e-175	490	99
NP_001280918	agamous-like MADS-box protein AGL1 [Malus domestica]	242	1	242	1	242	9	0	233	6.99e-173	484	99
XP_008342376	PREDICTED: agamous-like MADS-box protein AGL1 isoform X2 [Malus domestica]	243	1	242	1	243	8	1	234	8.83e-173	483	99
XP_017179299	PREDICTED: agamous-like MADS-box protein AGL1 isoform X1 [Malus domestica]	248	1	242	1	248	8	6	234	3.43e-172	482	99

UniProt -SwissProt-

Bit Score: <= 40 | 40 - 50 | 50 - 80 | 80 - 200 | 200 >=

ID 1 50 100 150

P29381 (http://www.uniprot.org/uniprot/P29381)

Q40885 (http://www.uniprot.org/uniprot/Q40885)

Q40168 (http://www.uniprot.org/uniprot/Q40168)

Q40872 (http://www.uniprot.org/uniprot/Q40872)

Q43585 (http://www.uniprot.org/uniprot/Q43585)

Subject ID	Description	Length	Q.start	Q.end	S.start	S.end	Mismatch	Gaps	Identity (%)	E-value	Bit score	Coverage (hsp) (%)
P29381	Agamous-like MADS-box protein AGL1 OS=Arabidopsis thaliana GN=AGL1 PE=1 SV=1	249	1	242	1	248	70	8	171	3.00e-116	335	99
Q40885	Floral homeotic protein AGAMOUS OS=Petunia hybrida GN=AG1 PE=1 SV=1	245	1	242	1	242	72	6	167	4.54e-113	327	99
Q40168	Floral homeotic protein AGAMOUS OS=Solanum lycopersicum GN=AG1 PE=2 SV=1	240	9	242	10	248	68	7	165	2.52e-111	322	96
Q40872	Floral homeotic protein AGAMOUS OS=Panax ginseng GN=AG2 PE=2 SV=1	241	3	242	4	242	76	3	162	1.62e-110	320	99
Q43585	Floral homeotic protein AGAMOUS OS=Nicotiana tabacum GN=AG1 PE=2 SV=1	240	9	242	10	248	67	7	166	1.41e-109	318	96

Query:Transcript

NCBI -nr-

Bit Score: <= 40 | 40 - 50 | 50 - 80 | 80 - 200 | 200 >=

ID 1 50 100 150 200 250 300 350 400 450 500 550 600 650 700 750 800 850 900 950 1000

BAM71400 (https://www.ncbi.nlm.nih.gov/protein/BAM71400)

AJW29041 (https://www.ncbi.nlm.nih.gov/protein/AJW29041)

NP_001280918 (https://www.ncbi.nlm.nih.gov/protein/NP_001280918)

XP_008342376 (https://www.ncbi.nlm.nih.gov/protein/XP_008342376)

XP_017179299 (https://www.ncbi.nlm.nih.gov/protein/XP_017179299)

Subject ID	Description	Length	Q.start	Q.end	S.start	S.end	Mismatch	Gaps	Identity (%)	E-value	Bit score	Coverage (hsp) (%)
BAM71400	transcription factor [Pyrus pyrifolia]	242	147	872	1	242	1	0	241	2.50e-177	500	22
AJW29041	AG-like MADS-box protein [Pyrus pyrifolia]	243	147	872	1	243	4	1	238	2.01e-173	491	22
NP_001280918	agamous-like MADS-box protein AGL1 [Malus domestica]	242	147	872	1	242	9	0	233	6.57e-171	484	22
XP_008342376	PREDICTED: agamous-like MADS-box protein AGL1 isoform X2 [Malus domestica]	243	147	872	1	243	8	1	234	7.61e-171	484	22
XP_017179299	PREDICTED: agamous-like MADS-box protein AGL1 isoform X1 [Malus domestica]	248	147	872	1	248	8	6	234	4.01e-170	482	22

UniProt -SwissProt-

Bit Score: <= 40 | 40 - 50 | 50 - 80 | 80 - 200 | 200 >=

ID 1 50 100 150 200 250 300 350 400 450 500 550 600 650 700 750 800 850 900 950 1000

P29381 (http://www.uniprot.org/uniprot/P29381)

Q40885 (http://www.uniprot.org/uniprot/Q40885)

Q40168 (http://www.uniprot.org/uniprot/Q40168)

Q40872 (http://www.uniprot.org/uniprot/Q40872)

Q43585 (http://www.uniprot.org/uniprot/Q43585)

Subject ID	Description	Length	Q.start	Q.end	S.start	S.end	Mismatch	Gaps	Identity (%)	E-value	Bit score	Coverage (hsp) (%)
sp|P29381|AGL1_ARATH	sp|P29381|AGL1_ARATH Agamous-like MADS-box protein AGL1 OS=Arabidopsis thaliana GN=AGL1 PE=1 SV=1	249	147	872	1	248	70	8	171	2.43e-114	335	22
sp|Q40885|AG_PETHY	sp|Q40885|AG_PETHY Floral homeotic protein AGAMOUS OS=Petunia hybrida GN=AG1 PE=1 SV=1	245	147	872	1	242	72	6	167	4.62e-111	327	22
sp|Q40168|AG_SOLLC	sp|Q40168|AG_SOLLC Floral homeotic protein AGAMOUS OS=Solanum lycopersicum GN=AG1 PE=2 SV=1	240	171	872	10	248	68	7	165	1.71e-109	323	21
sp|Q40872|AG_PANGI	sp|Q40872|AG_PANGI Floral homeotic protein AGAMOUS OS=Panax ginseng GN=AG2 PE=2 SV=1	241	153	872	4	242	76	3	162	9.39e-109	321	22
sp|Q43585|AG_TOBAC	sp|Q43585|AG_TOBAC Floral homeotic protein AGAMOUS OS=Nicotiana tabacum GN=AG1 PE=2 SV=1	240	171	872	10	248	67	7	166	1.13e-107	318	21

Similar sequences in Pyrus

Query:Protein

Chinese pear (*Pyrus brestschneideri*)

Bit Score: <= 40 40 - 50 50 - 80 80 - 200 200 >=

ID 1 50 100 150

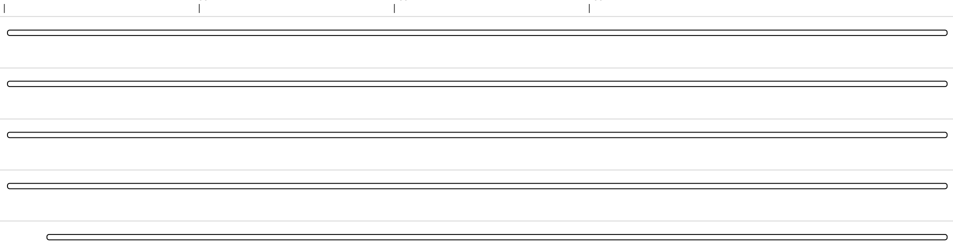

rna10647 (http://www.uniprot.org/uniprot/rna10647)

rna10644 (http://www.uniprot.org/uniprot/rna10644)

rna10645 (http://www.uniprot.org/uniprot/rna10645)

rna10646 (http://www.uniprot.org/uniprot/rna10646)

rna14870 (http://www.uniprot.org/uniprot/rna14870)

Show more ...

Subject ID	Description	Length	Q.start	Q.end	S.start	S.end	Mismatch	Gaps	Identity (%)	E-value	Bit score	Coverage (hsp) (%)
rna10647	rna10647 gene=LOC103947473 Dbxref=GeneID:103947473,Genbank:XM_009358383.1 Name=XM_009358383.1 gbkey=mRNA product=floral homeotic protein AGAMOUS-like%2C transcript variant X4 transcript_id=XM_009358383.1 gene_biotype=protein_coding	243	1	242	1	242	19	2	222	1.74e-163	452	99
rna10644	rna10644 gene=LOC103947473 Dbxref=GeneID:103947473,Genbank:XM_009358379.1 Name=XM_009358379.1 gbkey=mRNA product=floral homeotic protein AGAMOUS-like%2C transcript variant X1 transcript_id=XM_009358379.1 gene_biotype=protein_coding	243	1	242	1	242	19	2	222	1.74e-163	452	99
rna10645	rna10645 gene=LOC103947473 Dbxref=GeneID:103947473,Genbank:XM_009358380.1 Name=XM_009358380.1 gbkey=mRNA product=floral homeotic protein AGAMOUS-like%2C transcript variant X2 transcript_id=XM_009358380.1 gene_biotype=protein_coding	243	1	242	1	242	19	2	222	1.74e-163	452	99
rna10646	rna10646 gene=LOC103947473 Dbxref=GeneID:103947473,Genbank:XM_009358382.1 Name=XM_009358382.1 gbkey=mRNA product=floral homeotic protein AGAMOUS-like%2C transcript variant X3 transcript_id=XM_009358382.1 gene_biotype=protein_coding	243	1	242	1	242	19	2	222	1.74e-163	452	99
rna14870	rna14870 gene=LOC103951212 Dbxref=GeneID:103951212,Genbank:XM_009362520.1 Name=XM_009362520.1 gbkey=mRNA product=floral homeotic protein AGAMOUS-like%2C transcript variant X2 transcript_id=XM_009362520.1 gene_biotype=protein_coding	234	11	242	12	243	71	4	159	5.42e-111	319	95

Show more ...

European pear (*Pyrus communis*)

Bit Score: <= 40 40 - 50 50 - 80 80 - 200 200 >=

ID 1 50 100 150

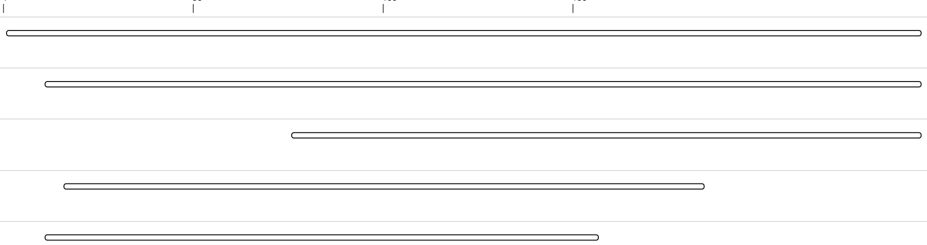

PCP023681.1 (http://www.uniprot.org/uniprot/PCP023681.1)

PCP044935.1 (http://www.uniprot.org/uniprot/PCP044935.1)

PCP005063.1 (http://www.uniprot.org/uniprot/PCP005063.1)

PCP032236.1 (http://www.uniprot.org/uniprot/PCP032236.1)

PCP031198.1 (http://www.uniprot.org/uniprot/PCP031198.1)

Show more ...

Subject ID	Description	Length	Q.start	Q.end	S.start	S.end	Mismatch	Gaps	Identity (%)	E-value	Bit score	Coverage (hsp) (%)
PCP023681.1	PCP023681.1 scaffold01895 14876 21592 + 1 687	243	1	242	1	228	16	16	211	1.08e-147	411	99

Subject ID	Description	Length	Q.start	Q.end	S.start	S.end	Mismatch	Gaps	Identity (%)	E-value	Bit score	Coverage (hsp) (%)
PCP044935.1	PCP044935.1	236	11	242	12	245	71	6	67.373	2.49e-107	310	95
PCP005063.1	PCP005063.1 scaffold00024 60267 62527 - 1 504	167	76	242	15	167	3	14	89.82	3.18e-106	304	69
PCP032236.1	PCP032236.1 scaffold00522 138682 151723 + . 1 1983	171	16	185	1	171	47	1	71.93	3.57e-82	258	70
PCP031198.1	PCP031198.1 scaffold01020 109835 116647 - 1 549	166	11	157	12	177	30	19	70.482	3.04e-79	236	60

Show more ...

Expression profiles

GPL13124 (https://www.ncbi.nlm.nih.gov/geo/query/acc.cgi?acc=GPL13124) Agilent National Institute of Fruit Tree Science/Fruit Genome Research Team -Pyrus pyrifolia-ver024595 (normarized by quantile method)

Pypy01331.1 has probe(s) for microarray experiments (for positions of the probes, please see 'JBrowse for microarray probes').
The prefix starting with NFR in the legend indicates name of probe.

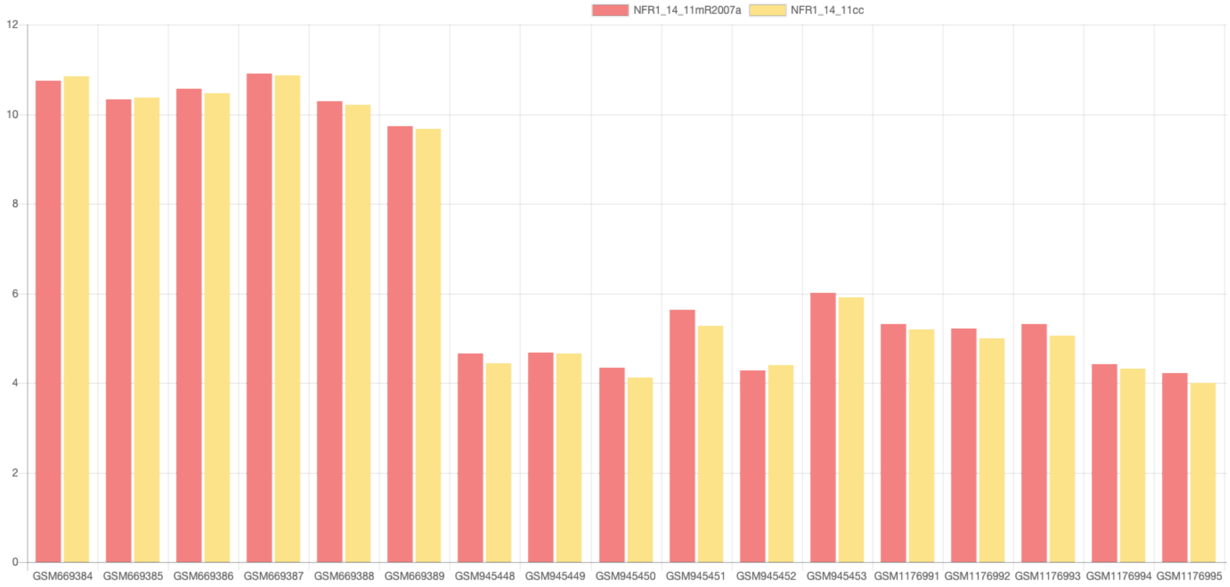

x-axis: Accession Organism Cultivar Organ Stage

Accession	Source	Organism	Cultivar	Organ	Stage	Title
GSM669384 (https://www.ncbi.nlm.nih.gov/geo/query/acc.cgi?acc=GSM669384)	2010_282_12 flower 1 day before bloom	Pyrus communis x Pyrus pyrifolia	La France x Housui hybrid	flower	1 day before bloom	024595_282_12_1DBF
GSM669385 (https://www.ncbi.nlm.nih.gov/geo/query/acc.cgi?acc=GSM669385)	2010_Housui flower 1 day before bloom	Pyrus pyrifolia	Housui	flower	1 day before bloom	-
GSM669386 (https://www.ncbi.nlm.nih.gov/geo/query/acc.cgi?acc=GSM669386)	2010_LaFrance flower 1 day before bloom	Pyrus communis	La France	flower	1 day before bloom	-
GSM669387 (https://www.ncbi.nlm.nih.gov/geo/query/acc.cgi?acc=GSM669387)	2010_LeConte flower 1 day before bloom	Pyrus hybrid cultivar	Bartlett hybrid (Bartlett is one parent)	flower	1 day before bloom	-
GSM669388 (https://www.ncbi.nlm.nih.gov/geo/query/acc.cgi?acc=GSM669388)	2010_Okusankichi flower 1 day before bloom	Pyrus pyrifolia	Okusankichi	flower	1 day before bloom	-

Show more ...

GPL9476 (https://www.ncbi.nlm.nih.gov/geo/query/acc.cgi?acc=GPL9476) Agilent custom array 60mer probes National Institute of Fruit Tree Science/Fruit Genome Research Team -Pyrus pyrifolia-18,862-ver017286 (normarized by quantile method)

Pypy01331.1 has probe(s) for microarray experiments (for positions of the probes, please see 'JBrowse for microarray probes').
The prefix starting with NFR in the legend indicates name of probe.

Supplementary Figure S1-6

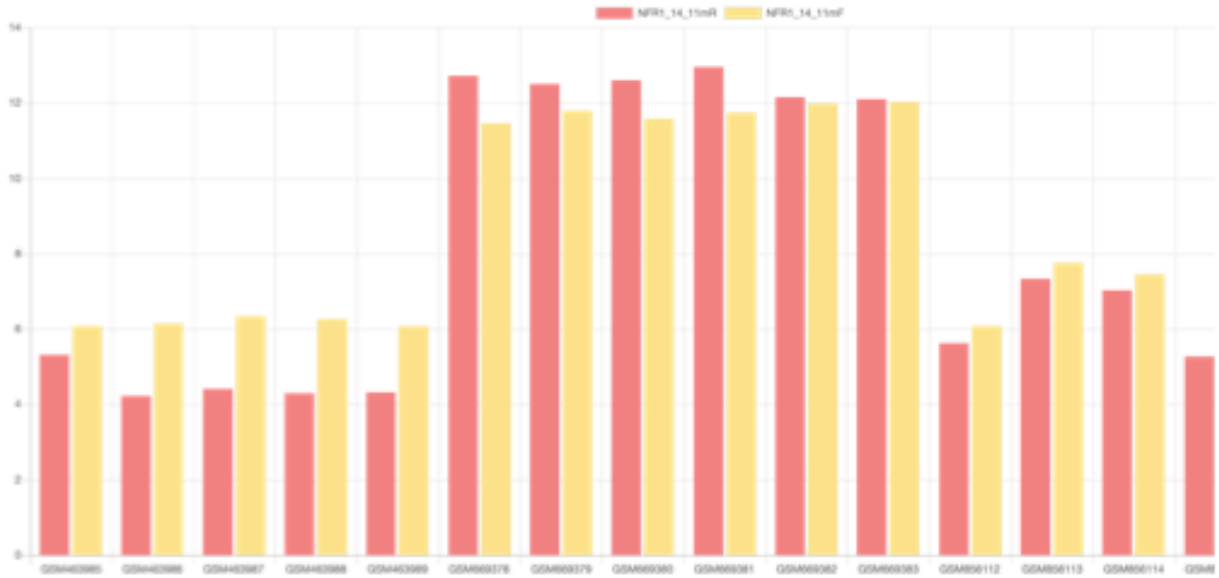

x-axis: Accession Organism Cultivar Organ Stage

Accession	Source	Organism	Cultivar	Organ	Stage	Title
GSM463985 (https://www.ncbi.nlm.nih.gov/geo/query/acc.cgi?acc=GSM463985)	Fruit 105 DAFB	Pyrus pyrifolia	Hosui Asian Pear	flesh	105 days after full bloom	Fruit 105 DAFB replicate1-3
GSM463986 (https://www.ncbi.nlm.nih.gov/geo/query/acc.cgi?acc=GSM463986)	Fruit 119 DAFB	Pyrus pyrifolia	Hosui Asian Pear	flesh	119 days after full bloom	Fruit 119 DAFB replicate1-2
GSM463987 (https://www.ncbi.nlm.nih.gov/geo/query/acc.cgi?acc=GSM463987)	Fruit 133 DAFB	Pyrus pyrifolia	Hosui Asian Pear	flesh	133 days after full bloom	Fruit 133 DAFB replicate1-3
GSM463988 (https://www.ncbi.nlm.nih.gov/geo/query/acc.cgi?acc=GSM463988)	Fruit 140 DAFB	Pyrus pyrifolia	Hosui Asian Pear	flesh	140 days after full bloom	Fruit 140 DAFB replicate1-3
GSM463989 (https://www.ncbi.nlm.nih.gov/geo/query/acc.cgi?acc=GSM463989)	Fruit 147 DAFB	Pyrus pyrifolia	Hosui Asian Pear	flesh	147 days after full bloom	Fruit 147 DAFB replicate1-3

Show more ...

JBrowse for microarray probes

The positions of the microarray probes in the reference sequences of Japanese pear are shown.

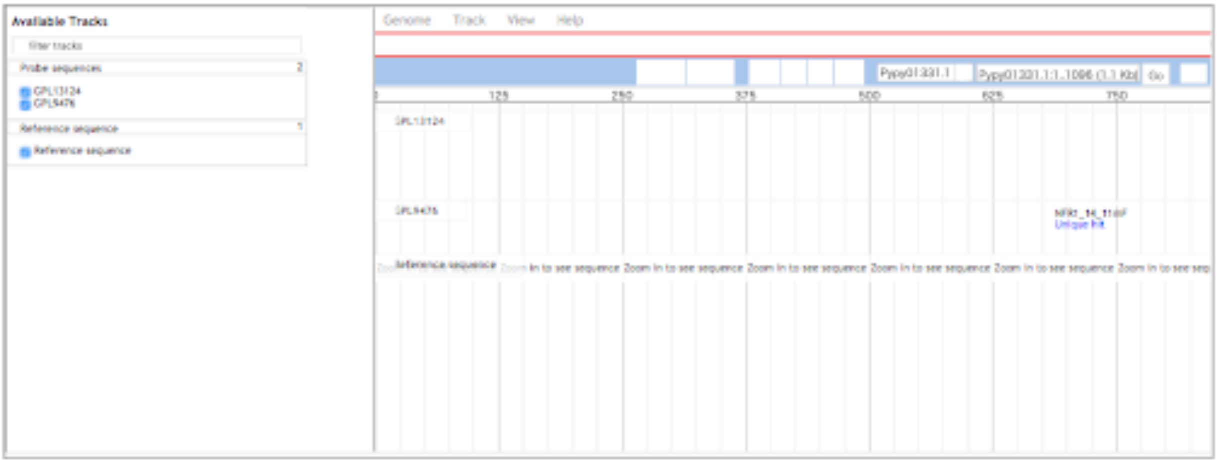

Sequences

cDNA

```
GAGTTTCCATTTCTGCAATTCCTCCTTCCcGTTGCCAATTGCAAACCCAATAGAAAAACT
CAAAGTCAAGAACTAGCTAACAGAAAAAaCCaCAATTCATCAATTTGgAGGGgTTTTTGC
CATTTTCCATCCttgcaattgcaaCAATGGAGTTCCCAAATCAAGCACCCGAGAGCTCcT
CCCAGAAAAAATTGGGAAGAGGCAAAATTGAGATTAAGCGGATCGAAAACACTACAAATC
GACAaGTaaCCTTCTGCAAaCGCCGCAACGGATTGCTTAAGAAAGCCTATGAATTGTCTG
TTCTTTGtGATGCTGAAGTtGCTCTTATCGTGTTCTCCAACCGTGGCCGCCTCTATGAGT
ATgCTAaCAACAGTGTTAGAGCAACAATCGACAGGTaCAAAAAaGCATGCtCTaATCCTA
CGAACGGTGGATCTGTTTCAGAAGCCAACACTCAGTTTTATCAGCAGGAAGCATCCAAAC
TGCGAAGACAGATCCGAGaaattCAGAATtcaaaCaggCATATACTgggTGAAGCTCTTA
GCaccTTGAACACCAAGGAACTGAAgAACCTAGAAGgAAGATTGGAGAAAGGAATCAGCA
GAATAAGATCCaagaaGAATGAAATgcTGTTTTCTGAAATCGAATTCATGCAAAAaaGGG
AGACCGAGCTGCAACACCACAACAATTTTCTGAGAGCAAAGATgGCTGAAAACGAGAGAG
AACAGCAGCAGCAAACACATATGATGCCGGGAACTTCCTACGATCAGTCGATGCCTTCGC
ATTCcTATGACAGGAACTTCCTCCCAGCgGTGATCTTGGAGTCCAACAATAACCATTACC
CTCACCAAGTCCAGACAGCTCTCCAACTTGTTTGAAATGCTGGACTGCCGTCTGATGTTC
TTCTATCgATATTATCTGATCTGTTTTCTTAAATCTATGAGATAATTGGTTGTGGTTTTA
TGTATATGGGAGAACCAGTTTGTTGTTCATGTTCTCCATAATATCAGAATTTTCTGTATT
AACTAGCTACTtTTTCAACTATGATGACAAGTAAaTTTGATTTCTCAACCTAATTAAAGT
TTCCATCTCTAAAAAA
```

Protein

```
MEFPNQAPESSSQKKLGRGKIEIKRIENTTNRQVTFCKRRNGLLKKAYELSVLCDAEVAL
IVFSNRGRLYEYANNSVRATIDRYKKACSNPTNGGSVSEANTQFYQQEASKLRRQIREIQ
NSNRHILGEALSTLNTKELKNLEGRLEKGISRIRSKKNEMLFSEIEFMQKRETELQHHNN
FLRAKMAENEREQQQQTHMMPGTSYDQSMPSHSYDRNFLPAVILESNNNHYPHQVQTALQ
LV*
```

Supplementary Figure S1. An example of a web page providing the detailed information of a transcript. The detailed information of the transcript ‘Pypy01331.1’ is shown here. This page is comprised of several sections: Summary, Functional annotations, Similar sequences in Pyrus, Expression profiles, and Sequences.

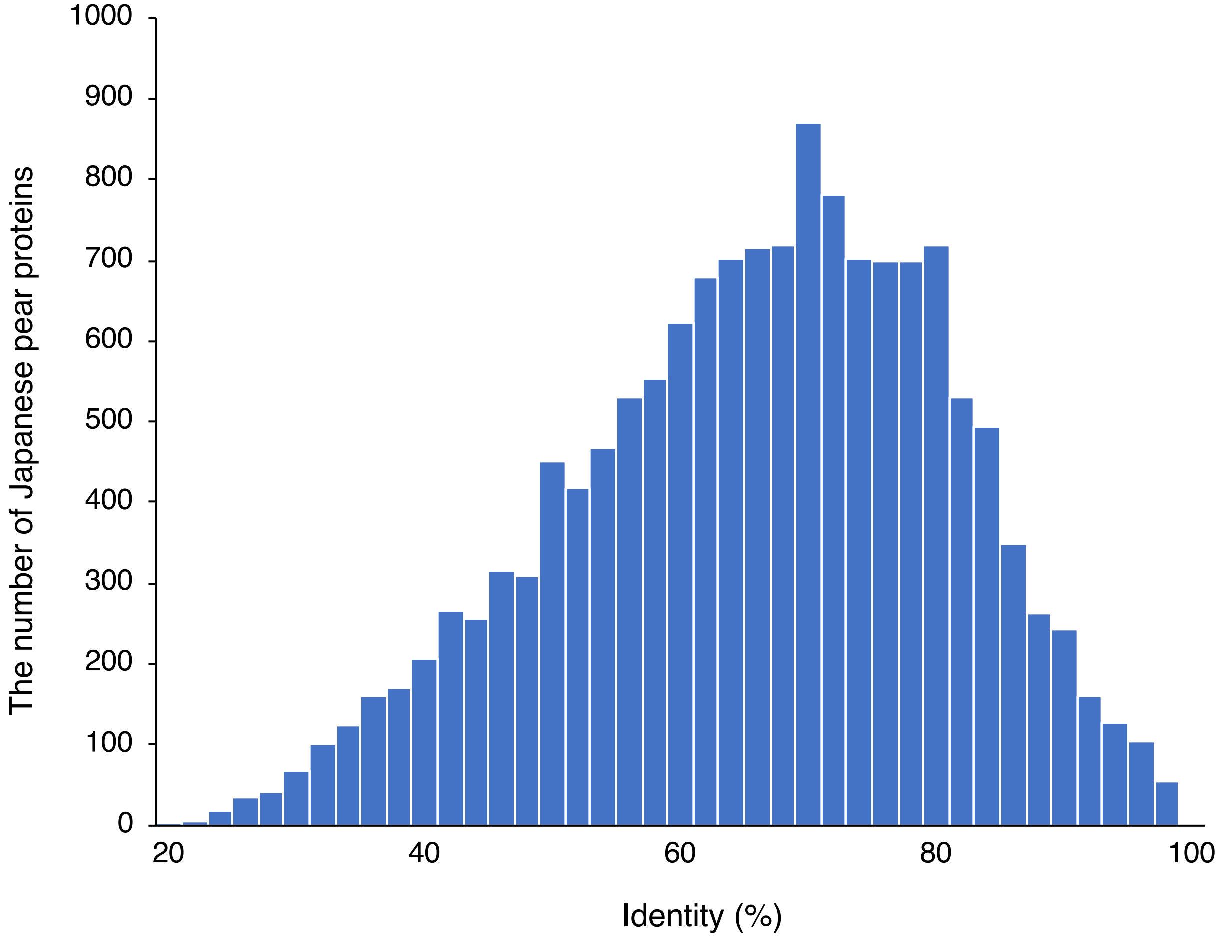

Supplementary Figure S2. Frequencies of sequence identities in pairwise alignments between Japanese pear and Arabidopsis proteins. Out of the complete ORFs beginning with start codons and ending at stop codons in the Japanese pear, 14,748 highly conserved protein sequences between Japanese pear and Arabidopsis were identified. The horizontal axis shows the identities of the pairwise alignments for the 14,748 highly conserved protein sequences.
